# On the Therapeutic Uses of Bromide of Ammonium

**Published:** 1866-12

**Authors:** Ira Hatch

**Affiliations:** Chicago, Ill.


ARTICLE XLIV.

### ON THE THERAPEUTIC USES OF BROMIDE OF AMMONIUM.

BY IRA HATCH, M.D., Chicago, Ill.

Read to the Illinois State Medical Society, June, 1866.

Soon after Dr. GIBBS' notice of this article, in the *London Lancet*, April, 1863, I commenced using it in various nervous affections connected with diseased and irritated mucous membranes, and in epilepsy. The effects were so striking, in some instances, that I have thought the record might be useful to the profession.

CASE I. Mrs. Eliza Gould, of Chicago, *æt.* 40. Chronic bronchitis, complicated with derangement of the liver and digestive organs. She had been subject to severe attacks of sick headache, violent vomiting, and palpitation of the heart. During these attacks, the difficulty of breathing was most distressing. Alarming fainting fits would precede each attack of vomiting, and dizziness, blindness, and numbness of the hands and feet would follow. Frequently, one or both sides would be paralyzed for several hours after these attacks. At one time, the paralysis of the right side lasted three weeks, but gradually wore off under the use of frictions to the spine and nux vomica internally; but it left her nervous, irritable, and extremely restless. There was more or less uterine irritation. She had never borne children. The throat and back part of the tongue had the appearance of fungous granulations. In order to allay nervous irritation, I gave her bromide of ammonium, in moderate doses, three times per day. It relieved the nervous irritation at once. She slept better; was more cheerful and comfortable when awake; she coughed less, and complained less of numbness of the extremities; and, was far less apprehensive of approaching danger. In short, the sedative effects of the remedy were very strongly marked.

CASE II. Mrs. Mary E., of Chicago, *æt.* 39. Uterine irritation and leucorrhœa. This woman had been married 15 years; had never borne children; was of a nervous temperament; and had been always a victim of dysmenorrhœa, lucorrhœa, and dyspepsia, which latter complaint had become, latterly, much aggravated, being attended by vomiting and other unequivocal symptoms of pregnancy. In March, 1864, the seventh month of her pregnancy, she had whooping-cough, which was so severe that it brought on premature labor. Her labor, as well might be expected, was tedious and painful, and her recovery slow and unsatisfactory. She lost her appetite; became excessively nervous; had fainting fits of a hysterical character; and laughed and cried in the same breath. She would not be left alone a moment, for fear of dying. Yet, she had no fever; her pulse, respiration, and skin natural; she had no tenderness of the abdomen, but the bowels were obstinately costive—every evacuation prostrated her and aggravated all her nervous symptoms. She did not bear opium well, and nervines were of no account. I had noticed, during her labor, that the os uteri was extremely sensitive, and this led me to attribute her tedious convalescence to uterine irritation. She was put upon the use of bromide of ammonium, in 4 gr. doses every six hours. The effect was most gratifying. She slept quietly the first night, which she had not done during her confinement; she had no more fainting fits or palpitation, and made a quick and perfect recovery. The bromide relieved the constipation, she has enjoyed good health since, and has had no dysmenorrhœa.

CASE III. Epilepsy. Jennie Warren, of Chicago, *æt.* 7. She had averaged three fits daily for nearly six years; had become extremely sensitive and irritable; recoiled at every touch, and started at every sound. Her memory was much impaired, and her countenance had an idiotic expression; her gait was unsteady, and the left arm was drawn out of shape and partially paralyzed. This case was certainly very unpromising. Her parents had ceased making any efforts in her behalf, having been repeatedly told that the case was hopeless. Being in attendance upon another child in the family, I casually made the remark that I would try the bromide of ammonium, as I had lately seen it recommended by high authority. The mother caught at the suggestion, and immediately commenced the use of it, in doses as large as the stomach would bear without vomiting, three times per day. She took the remedy about fourteen months in all, and has not had a fit for nearly two years. The convulsions began immediately to abate, both in frequency and severity; she recovered her consciousness much sooner after each fit, and soon became less fretful and sensitive. In less than a week, her mother, who watched her with intense anxiety, became satisfied that there was an improvement in the general appearance of her child, and persevered in the use of the medicine with a zeal and determination worthy of the cause.

She did not, like many parents, expect and demand too much at once; although the fits continued, she did not drop the remedy, but felt confident of ultimate success. The fits were brought on several times by indigestion. With a nurse less observing and less persevering, we should have failed of success, and the bromide, as in other unskilful or unfaithful hands, would have lost its credit.

I am satisfied that this remedy not only calms the irritability of the nervous centres, but it allays the irritation of the nervous extremities, thus removing both the predisposing and exciting causes of epilepsy. It strengthens the machinery and at the same time lessens the friction. The child is apparently cured of a most formidable disease of long standing; the morbid connexion between the irritability of the digestive organs and the excitable cerebro-spinal axis seems to be broken up; the effects of the friction of an irritable mucous membrane acting upon a sensitive nervous system seem to have yielded to the soothing effects of the bromide of ammonium. Epilepsy, arising from some organic lesion of the spinal centre, without any eccentric or outside irritation, would, most likely, be incurable. But these cases are probably very rare. Most cases evidently arise, at first, from outside irritation applied to the extremities of the nerves, which irritation is carried up to the centres and constantly accumulates there, till it explodes or culminates in a convulsion.

Case IV. Epilepsy. Miss Hannah F., Chicago., *æt.* 16. This patient is now under treatment, having recently had a fit. She has a nervous temperament; is naturally irritable; had convulsions in infancy; and had an aunt die with epilepsy. The nervous centres are weak and excitable. There is, in her case, a strong hereditary predisposition to epilepsy. The exciting cause of the convulsions, in every instance, has been disturbed indigestion. Constipation, yellow skin, nausea, and pain in the epigastrium have been the usual precursors of a convulsion. She becomes forgetful, flighty, peevish, and melancholy before a convulsion. These latter symptoms have always been controlled, and the fit prevented, by the bromide, when properly attended to. Twice, within the last six months, the treatment has been neglected, and, under excitement, the fits have occurred. The first was under the excitement of a Christmas tree, at home; and the second, recently, was under the excitement of a strawberry festival. This case will probably recover by close attention to the digestive organs, and by controlling nervous excitement by the bromide. The convulsions do not seem to be influenced by the menses, which are not disturbed. It is remarkable how soon the premonitory symptoms of a convulsion will yield under the influence of this potent remedy.

CASE V. Epilepsy. George Beers, Chicago. *æt.* 11. This lad had epileptic fits about six years. At first, they were very mild, they seemed to be mere aura epileptica, and occurred not oftener than once a-month; but they gradually increased in force and frequency, until they became very severe and often repeated. He was under homœopathic treatment four or five years, and tried various quack remedies, always with injury. His father paid a quack, in Cincinnati, one hundred dollars to cure him, who nearly killed him. In this condition, he came under my care. He was of a lymphatic temperament, with scrofulous tendencies. He was put upon the use of cod liver oil and iron, and the fits were controlled by the bromide of ammonium in 10 gr. doses, three times per day. This was in June, 1865. The fits gradually lessened in violence and frequency till the 1st of September, since which time he has had none. The alteration and improvement in his countenance and intellectual faculties are most striking. He now seems to be entirely cured. This case and that of the Warren girl, are two of the most remarkable triumphs in medicine; as gratifying to the profession as they were mortifying to quackery and empiricism. I have two other cases of epilepsy under treatment, with every prospect of success, but, as the convulsions occur occasionally, I do not report them at this time.

CASE VI. Metro-gonorrhœa. Miss R., *æt.* 21. A young scamp had given her gonorrhœa, and attempted to cure it by injections of tr. mur. iron, and had produced a severe inflamma-

46

tion of all the organs of generation, involving also the urethra and the neck of the bladder. These symptoms were combated by leeches and an active antiphlogistic course of treatment. The urgent symptoms yielded somewhat to this course, but the congestion and pain in the uterus, together with the gonorrhœal discharge, continued. Some of the usual anti-gonorrhœal remedies were tried, but the stomach, which was very irritable, rejected them all. The patient was excessively nervous and scarcely slept at all. With a view simply to make her sleep, which narcotics failed to do, I gave her 15 gr. doses of bromide of ammonium every four hours. She slept soundly the first night; the pain and swelling of the uterus gradually yielded; and she recovered entirely, without any other medicine. It not only relieved the gonorrhœa, but the irritation of the stomach quickly subsided, and the appetite returned.

I have used the bromides of potassium and ammonium, in a great variety of minor cases, to allay irritation and procure sleep in children, and generally with satisfactory results.

NOTE.—Cases I. and II. have been published in the CHICAGO MEDICAL EXAMINER.

